# 

**Published:** 1910-10

**Authors:** 


### BOOKS RECEIVED

A Manual of Hygiene and Sanitation. By Seneca Egbert, M.D., Dean and Professor of Hygiene in the Medico-Chirurgical College, Philadelphia. Fifth edition, thoroughly revised. 12mo, 508 pages, with 97 illustrations. Lea & Febiger, Philadelphia and New York, 1910. (Cloth, $2.25, net.)

A Manual of Obstetrics. By A. F. A. King, M.D., Professor of Obstetrics and Diseases of Women in the Medical Department of the George Washington University, Washington, D. C., and in the Medical Department of the University of Vermont, etc. Eleventh edition. 12 mo, 713 pages, with 341 illustrations and three colored plates. Lea & Febiger, Philadelphia and New York, 1910. (Cloth, $2.75, net.)

Physical Examination and Diagnostic Anatomy. By Charles B. Slade, M.D., Instructor in Physical Diagnosis, University and Bellevue Hospital Medical College, New York. 12 mo, of 146 pages, illustrated. Philadelphia and London: W. B. Saunders Company, 1910. (Cloth, $1.25 net.)

Dawn of the Fourth Era in Surgery and Other Short Articles. By Robert T Morris, M.D., Professor of Surgery, New York Post-Graduate Medical School and Hospital. 12mo, of 145 pages. Philadelphia and London: W. B. Saunders Company, 1910. ($1.25 net.)

Hydrotherapy: A Treatise on Hydrotherapy in general; its application to special affections; the Technic or Processes Employed; and use of Waters Internally. By Guy Hinsdale, A.M., M.D., Lecturer on Climatology, Medico-Chirurgical College of Philadelphia. Octavo of 466 pages, illustrated. Philadelphia and London: W. B. Saunders Company, 1910. (Cloth, $3.50 net.)

Veterinary Anatomy. By Septimus Sisson, S.B., V.S., Professor of Comparative Anatomy, Ohio State University, College of Veterinary Medicine. Octavo of 826 pages, 528 illustrations. Philadelphia and London: W. B. Saunders Company, 1910. (Cloth, $7.00; half morocco, $8.50 net prices.)

The Principles of Pathology. Volume I, General Pathology. By J George Adami, M.A., M.D., LL.D., F.R.S., Professor of Pathology in McGill University, Montreal. Second edition, thoroughly revised. Octavo, 1027 pages, with 329 engravings and 18 plates. Lea & Febiger, Publishers, Philadelphia and New York, 1910. (Cloth, $6.00, net.)

Anatomy, Descriptive and Applied. By Henry Gray, F.R.S., late lecturer on anatomy at St. George's Hospital, London. Eighteenth edition revised by Edward Anthony Spitzka, M. D., Professor of Anatomy in the Jefferson Medical College of Philadelphia. Imperial octavo, 1496 pages, with 1208 engravings. Lea & Febiger, Publishers, Philadelphia and New York. (Cloth, $6.00; leather, $7.00 net prices.)

Lippincott's New Medical Dictionary. By Henry W. Cattell, A. M., M. D., Editor of International Clinics. Octavo, pp. 1108. Illustrated. Philadelphia and London: J. B. Lippincott Co. (Limp leather, thumb index, $5.00.)

Normal Histology. By George A. Piersol, M. D., Sc. D., Professor of Anatomy in the University of Pennsylvania. Eighth edition. Octavo, pp. 426. Illustrated. Philadelphia and London: J. B. Lippincott Co. (Cloth, $3.50.)

Difficult Labor. By G. Ernest Herman, M. B. Lond., Consulting Obstetric Physician to the London Hospital. 12 mo, pp. 559. Illustrated. New York: William Wood & Co., 1910. (Cloth, $2.50.)

Diseases of the Colon and their Surgical Treatment. By P. Lockhart Mummery, F. R. C. S. Eng., Jacksonian Prizeman and late Hunterian Professor, Royal College of Surgeons, London. Small octavo, pp. 330. Illustrated. New York. William Wood & Co. 1910. (Cloth, $3.25.)

Manual of Obstetrics. By A. F. A. King, A M., M. D., Professor of Obstetrics in the Medical Department of the George Washington University, Washington, D. C. Eleventh edition. 12 mo pp. 733. Illustrated. Philadelphia and London: Lea & Febiger. 1910. (Cloth, $7.75.)

Vaccine Therapy. Its Theory and Practice. By R. W. Allen, M. D., London. Third edition. Small octavo, pp. 287. Philadelphia: P. Blakiston's Son & Co. 1910. (Cloth, $2.00.)

Symptomatic and Regional Therapeutics. By George Howard Hoxie, M D., Professor of Internal Medicine in the School of Medicine, University of Kansas, Lawrence. Octavo, pp. 517. Illustrated. New York and London: D. Appleton & Co. 1910. (Cloth, $4.00.)

Transactions of the thirty-second annual meeting of the American Laryngological Association held at Washington, D. C., May 3-5, 1910. James E. Newcomb, M D., secretary.

Progressive Medicine, Vol. 12, September, 1910. A Quarterly Digest of Advances, Discoveries and Improvements in the Medical and Surgical Sciences. Edited by Hobart Amory Hare, M. D., Professor of Therapeutics and Materia Medica in the Jefferson Medical College of Philadelphia and Leighton F. Appleman, instructor in therapeutics at the same college. Philadelphia and New York: Lea & Febiger. (Per annum, paper bound, $6.00; cloth, $9.00.)

Cystoscopy as Adjuvant in Surgery, with an Atlas of Cystoscopic Views and Concomitant Text for Physicians and Students. By Staff Surgeon Dr. O. Rumpel, Lecturer in Surgery at the University of Berlin. Authorized English Translation by P. W. Shedd, M. D., New York. Octavo, pp. 131. With illustrations in color on 36 plates and 22 textual figures. New York: Rebman Company. (Half leather, $8.50.)

